# DiffCoRank: A Comprehensive Framework for Discovering Hub Genes and Differential Gene Co-expression in Brain Implant-Associated Tissue Responses

**DOI:** 10.21203/rs.3.rs-6647970/v1

**Published:** 2025-05-20

**Authors:** Anirban Chakraborty, Erin K Purcell, Michael G Moore

## Abstract

**Background:** Brain implants have significant potential for therapeutic applications and neuroscience research, but complex tissue responses often compromise their long-term stability. To address this challenge, differential coexpression analysis can be used to identify key molecular regulators involved in brain implant responses. **Results:** We developed DiffCoRank, an integrated framework that improves differential coexpression analysis by integrating the techniques of RNA-Seq data preprocessing, gene filtering, correlation-based module identification, and network analysis to discover differentially coexpressed gene clusters. A key innovation of our approach is false discovery rate (FDR)-based selection of strongly connected genes (SCGs), by which we improve detection of strong coexpression patterns that otherwise could be lost to spurious correlations. To enhance the identification of different modules, we employ a hybrid clustering technique that combines uniform manifold approximation and projection (UMAP) with density-based spatial clustering of applications with noise (DBSCAN). We propose a multicriteria hub gene ranking system incorporating network centrality metrics such as degree, closeness, betweenness, and eigenvector centrality to prioritize biologically relevant genes. **Conclusions:** Our method successfully identified important gene modules linked to stress responses, immunological regulation, and axonal pathfinding from the RNA-Seq data of implanted rat brain tissue. Furthermore, we compared our results to those of other existing coexpression analysis frameworks, showing that our method successfully identifies unique regulatory processes and consistent coexpression patterns. Our research offers novel insights into the molecular processes that explain implant-tissue interactions and possible approaches to improve the robustness and biocompatibility of brain interfaces.

## 1 Introduction

Brain implants have gained popularity in research and clinical areas, with applications including brain-computer interfaces (BCI), neuroprosthetics, and neuromodulation. These implants are used for the acquisition of brain signals and/or the stimulation of neural circuits [1]. This technology is increasingly used to treat clinical conditions such as Parkinson's disease, essential tremor, dystonia, intractable epilepsy, and chronic pain [2, 3]. Despite their potential, these implants trigger biological reactions that may compromise their long-term stability and effectiveness. These implants typically cause glial scarring, inflammation, and other cellular modifications due to the foreign body response of the brain, which leads to impairment of the electrode's capacity to record or stimulate brain activity [4]. This results in recording instability, shifting stimulation thresholds, and altered neural excitability [5] [6].

Understanding the biological response to electrode implantation at the molecular level is necessary to optimize electrode performance and design [7, 8]. Advanced techniques such as RNA sequencing (RNA-seq) provide a detailed analysis of gene expression changes at the implant site, facilitating the identification of differentially expressed genes associated with tissue responses. Thompson et al. (2021) [9] used RNA-seq to study spatiotemporal gene expression patterns around implanted silicon microelectrode arrays in the rat motor cortex. The tissue within 100 µm of the electrode was compared to naïve tissue, and the study revealed 157 differentially expressed (DE) genes, with 94 DE genes between interfacial and distal (500 µm) tissue. These DE genes included those linked to inflammation, gliosis, synaptic plasticity, and neural structure. These genes may help us understand how neurons and glial cells react to implanted devices [9]. Subsequent work verified the expression of RNA-seq-identified genes at the protein level via immunohistochemistry [10]. Similarly, researchers identified chronic neuroinflammatory markers and extracellular matrix remodeling genes, including *C3*, *MMP12*, and *SPP1*, in mice implanted with microelectrodes [11]. These findings, which are supported by protein-level validation via immunohistochemistry, indicate the ongoing role of molecular pathways in modulating the chronic response to intracortical implants. By identifying the genes responsible for the foreign body reaction, these methods can identify potential molecular targets to enhance the stability and biocompatibility of neural implants.

Gene expression analysis provides a complete picture of the molecular pathways triggered in response to the implanted device, providing insights into the underlying mechanisms that drive tissue reactions [12]. The study of gene expression near the implant site is important because it captures a broad repertoire of effects of implantation, including acute and chronic changes in cellular activity, synaptic remodeling, neuroinflammatory processes, and neuroplasticity. If these molecular alterations can be examined, we can identify important regulatory routes and potential biomarkers

[13] to improve the biocompatibility of neural implants and reduce tissue reactions [9]. However, the resulting datasets are complex and large, making it challenging to derive biologically useful information. Weighted gene coexpression network analysis (WGCNA) addresses these issues by building networks that identify modules of coexpressed genes via pairwise correlations. These modules were examined for differential coexpression, which revealed dynamic changes in gene regulatory networks across situations [14]. For example, by utilizing the topological overlap metric, WGCNA makes it easier to find weak correlation patterns, allowing for the identification of biologically significant clusters under varying experimental conditions. Advanced approaches, such as differential coexpression analysis (DiffCoEx)[15], extend the concept by identifying changes in the correlation structure of modules under different experimental conditions, providing further information about how transcriptional networks reorganize in response to implantation. These methodologies ensure that transcriptomic assays provide actionable insights into the molecular mechanisms underlying device-tissue interactions and how they can be modulated to improve biocompatibility. Gene ontology and network analyses are useful solutions for these issues.

In this study, we examined gene expression data from near-implanted electrode sites in rat brains via RNA sequencing (RNA-Seq) to understand how tissue responses affect the biocompatibility of neural implants. Using RNA-Seq data from 17,317 genes in 36 samples taken near the implant ($\leq$ 100 μm) and farther from the implant ($\sim$ 500 μm), we identified genes with significant expression differences between these regions. In our previous study [13], DiffCoEx [15] was used to determine the differentially coexpressed gene modules between tissue samples collected at different distances from implanted electrode sites. In this method, protein-coding genes were analyzed, and genes with low counts were excluded, followed by hierarchical clustering of genes. Using a topological overlap matrix, hierarchical clustering was performed to identify modules with distinct correlation structures based on adjacency differences between conditions. An eigengene-based approach was then applied, where principal component analysis (PCA) was used to compute the eigengene (the first principal component) for each module. The gene with the highest loading on this eigengene was identified as the hub gene, representing the key regulatory gene in that module. In the present study, we extend this approach in multiple ways. First, we employ a false discovery rate (FDR) threshold, enabling us to focus on genes with statistically significant differential coexpression. Second, we build upon the methods used to identify hub genes by incorporating multiple statistical criteria, including network centrality measures such as degree and closeness, in addition to the previously used eigen-centrality metric. Furthermore, we explore alternative clustering approaches and visualize the resulting gene relationships using uniform manifold approximation and projection (UMAP). Finally, we assessed the results of both our dataset and a previously published, unrelated dataset of mutant (Eker) rats exposed to carcinogens and wild-type rats exposed to the same carcinogens [16]. These techniques extend upon the growing use of advanced techniques to understand key biomarkers of device-tissue interactions in the brain via transcriptomic data analysis.

## 2 Methodology

### 2.1 Description of the dataset

The data used in this analysis are publicly accessible at NCBI Sequence Read Archive (SRA) under BioProject ID **PRJNA1089183** (https://www.ncbi.nlm.nih.gov/sra/PRJNA1089183) and have been reported previously [17]. The dataset is reported here under an open-access Creative Commons license (NCBI BioProject: **PRJNA1089183**).

Briefly, the motor cortex (M1) of adult Sprague–Dawley rats was stereotaxically implanted with single-shank, 'Michigan'-style probes (16-channel A1 $\times$ 16, 3 mm, 15 $\mu$m thick, 703 $\mu m^2$ site size, 100 $\mu$m site spacing, Neuronexus Technologies), as previously reported [9]. All surgical procedures described were authorized by the Michigan State University Animal Care and Use Committee. As described, brain tissue adjacent to the implant (interfacial, within 100 µm) was extracted using laser capture microscopy from cryosections collected following euthanasia and perfusion fixation. Distal tissue of an approximately equivalent total surface area was extracted from ($\sim$ 500 µm) away from the implant site. RNA was extracted and sequenced as described [9].

### 2.2 Data analysis and differential co-expression pipeline

Genes were clustered based on the differential gene-to-gene correlation between the two conditions. We began with RNA-seq data consisting of raw counts and rlog-normalized counts from 17,317 genes identified in 36 samples. The samples are divided into two conditions: 'near' (within 100 microns of the device) and 'far' (approximately 500 microns from the device). For preprocessing, we queried the BioMart Ensembl database [18] via the REST application programming interface (API) to obtain the gene name, biotype, and gene length for each gene. We filtered the genes to include those that met the following criteria: (1) a minimum of 100 total raw counts across samples; (2) appeared in at least three samples per condition; (3) protein-coding; (4) gene length of at least 100 base pairs (to avoid snippets); and (5) an identified external gene name. After filtering, 69% or 11,400 genes remained.

Following filtering, we devised an approach to focus on genes with statistically significant correlations. Traditional methods for assessing gene coexpression frequently involve calculating pairwise correlation coefficients for all genes in a certain dataset, followed by significance testing to discover correlations above a predetermined threshold [19, 20]. The original DiffCoEx methodology [15] is intended to detect module-level differential coexpression patterns, in which clusters of genes show coordinated shifts in correlation under different conditions. It first defines modules by clustering algorithms, after which it examines the differential correlations inside and across these modules.

This method excels at detecting large-scale, organized changes in network topology, but it may miss small changes at the single-gene level. In contrast, our methodology employs a gene-centric approach. Instead of starting with modules, we first computed Spearman correlation coefficients [21] and p-values for the filtered genes under both near and far conditions. We then combined the sets of p-values from both conditions and used the Bonferroni method to map the p-value onto the false discovery rate (FDR). This approach minimizes false positives that could obscure biologically meaningful patterns without resorting to computationally intensive permutation steps, as followed in the DiffCoEx Paper [15]. Using FDR $< 10\%$ or $< 50\%$ as thresholds, both yield a nearly identical set of outliers of strongly connected genes (SCGs) (Figure 2). Genes with more than 200 correlations at 50% FDR and more than 20 at 10% FDR—were selected as strongly connected genes (SCGs) for downstream analysis. Among the 11,400 filtered genes, approximately 1,722 were highly correlated (around 15%). The remaining genes form a bulk group with low numbers of strong correlations. For the remainder of our analysis, we focused on the subset of SCGs as candidates for meaningful differential coexpression modules.

Following the DiffCoEx pipeline [15], the adjacency and topological distance between the SCGs are calculated. Our adjacency matrix evaluates the absolute differences in gene-gene correlations between conditions to detect differential co-expression, in contrast to conventional co-expression networks that estimate gene similarity in a single condition. This emphasizes changes in network structure that may be biologically significant to spatial tissue responses. In this paper, adjacency is defined by $A = |C_1 - C_2|$, where $C_j$ is the correlation matrix under condition $j = 1, 2$ ($j = 1$ represents the near device condition, and $j = 2$ represents the far device condition). Although the possible range of values for an element of a matrix $A$ is $[0, 2]$, we set a hard cap of 1, meaning that any change in the correlation value of 1 or more results in the maximum adjacency. By capping adjacency values at 1, we aimed to prevent excessively large correlation differences from distorting the network structure. Furthermore, changes in correlation values greater than one are uncommon and may represent statistical anomalies or data artifacts rather than significant biological abnormalities. By placing a hard cap at one, we reduce the impact of these outliers, focusing our research on accurate and interpretable co-expression variations [22]. To devise the topological distance matrix, we adopted the method described in [15], which utilizes the second-order topological overlap matrix (TOM) distance. This technique minimizes the distance between genes that are connected to a similar set of genes, highlighting the clustering of genes with similar coexpression patterns. The TOM-based distance matrix ensures that genes with common neighbors in the coexpression network are more efficiently clustered together, resulting in stronger and more biologically relevant clusters [23, 24].

#### 2.3 Clustering and quality assessment

We utilized uniform manifold approximation and projection (UMAP) to visualize the resultant clusters in a lower-dimensional space, providing a comprehensible representation of the high-dimensional gene expression data. UMAP is effective for showing complicated relationships in data while keeping the local structure in embeddings. In our work, we utilized UMAP to project the gene modules into a 2D space, highlighting clusters with different colors and labels. In our analysis, we tuned key UMAP parameters (number of nearest neighbors, minimum distance, and random state) to maximize cluster separation and maintain the local topology of the data. To assess the quality of the clustering results generated via UMAP and DBSCAN, we used two complementary metrics: the silhouette score [25] and the Davies-Bouldin index [26]. These measures enabled us to evaluate the distance between clusters and the compactness of individual clusters, respectively, providing an option for finding the best clustering configurations. Specifically, we examined the top nine distinct clustering

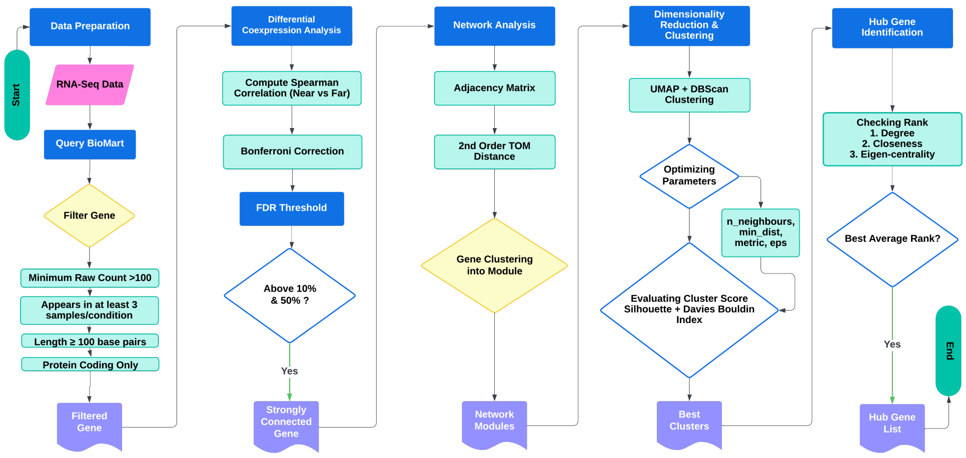

**Fig. 1 Overview of the computational workflow for hub gene identification.** Key steps include differential coexpression analysis, network clustering via TOM, UMAP+DBSCAN-based dimensionality reduction, and centrality-based ranking to identify biologically relevant hub genes.

solutions (Figure 3) created by modifying factors such as the number of neighbors (`n_neighbors`), minimum distance (`min_dist`) and DBSCAN parameters (`eps` and `min_samples`). Clusters with higher silhouette scores demonstrated distinct and well-separated groupings of data points. In contrast, lower silhouette scores were observed in configurations where cluster boundaries were less distinct, reflecting overlapping or ambiguous groupings. The Davies-Bouldin index (DBI), which measures the ratio of intracluster compactness to intercluster separation, provides additional insights into cluster quality. Lower DBI values indicate more compact clusters with better separation between them. In contrast, higher DBI values were associated with fewer distinct clusters and greater variability in cluster dispersion. The combination of these metrics enabled us to identify optimal clustering configurations, balancing well-separated clusters with compact groupings.

To assess clustering performance, we standardized the silhouette score and Davies-Bouldin index to a [0, 1] scale so that we could compare them directly. We initially performed a comprehensive grid search across all parameter combinations to refine the scoring method, visually inspected the resulting clusters, and adjusted the weights (0.45 for the silhouette score and 0.55 for the Davies-Bouldin index) to prioritize well-defined, compact clusters with clear boundaries. This weighting reflected the significance of compact, well-defined clusters in our research.

We then analyzed the clusters via several standard network centrality measures to identify hub genes within each module. Using the *networkx* package [27] (v1.10) in Python v3.8, we computed degree, closeness, betweenness, and eigenvector centrality. Degree centrality refers to the number of direct connections (edges) that a gene has within a module. Genes with a high degree of centrality are used for hub gene calculation and are central to the module structure [28]. Closeness centrality indicates how close a gene is to all other genes in the network, with higher closeness centrality indicating that the gene will have a more powerful direct influence on other genes [29]. Betweenness centrality measures how frequently a gene works as a bridge along the shortest path between other genes in the network [30, 31]. Eigenvector centrality quantifies the degree to which a gene is linked to other highly connected genes in the network. It ranks genes according to their overall influence and relevance in the biological pathway or system to which they belong [32]. Based on the average ranks of each metric, we identified the hub genes for the network.

### 2.4 Assessments of the approach

To assess our approach, we applied our method to an additional dataset (Gene Expression Omnibus GEO GSE5923 - Eker Dataset) [16], which includes Affymetrix gene expression profiles of the renal cortex outer medulla in wild-type and Eker rats treated with carcinogens. This dataset was analyzed in the original differential coexpression paper published [15]. Additionally, we implemented an alternative pipeline, the gene whole coexpression network analysis (GWENA) package [33], on our data and compared the results to our differential coexpression strategy. The GWENA package focuses on building scale-free networks and detecting gene modules using hierarchical clustering. It connects modules to biological functions through multi-database enrichment and identifies hub genes with topological metrics. Our approach focuses on gene-level filtering, UMAP-DBSCAN clustering, and direct hub gene detection without external enrichment analysis.

## 3 Results

In this study, differential coexpression analysis was performed to identify the hub gene modules exhibiting significant differences in coexpression patterns between "near" and "far" tissue samples in the vicinity of an implanted microelectrode. The analysis included data preparation, gene filtration, correlation calculation, identification of strongly correlated genes, dimensionality reduction via UMAP, density-based clustering, and module analysis. To ensure that the genes with sufficient representation are included in the actual analysis and to determine the appropriate parameter for filtration, we plotted the genes expressed under both "near" and "far" conditions to visualize gene expression consistency. The outcomes and intermediate steps of this analysis are visually summarized in Figure 2.

Following the removal of weak correlations, we computed the adjacency matrix as the absolute difference in correlation coefficients between near and far conditions (threshold $< 0.03$). Figure 2(e) displays logarithms of adjacent values derived from the difference in correlation coefficients between conditions. This histogram shows the distribution of log-transformed adjacency values ($log(A)$), with most values ranging between -3 and -1, indicating that most gene pairs experience minor changes in correlation between conditions. The frequency peaks around log(A) = -1, with over 90,000 gene pairs falling within this range, indicating prevalent minor differential coexpression. The left tail ($\log(A) < -4$), where frequencies decrease below 10,000, indicates that fewer gene pairs have larger changes. This pattern indicates that while most gene

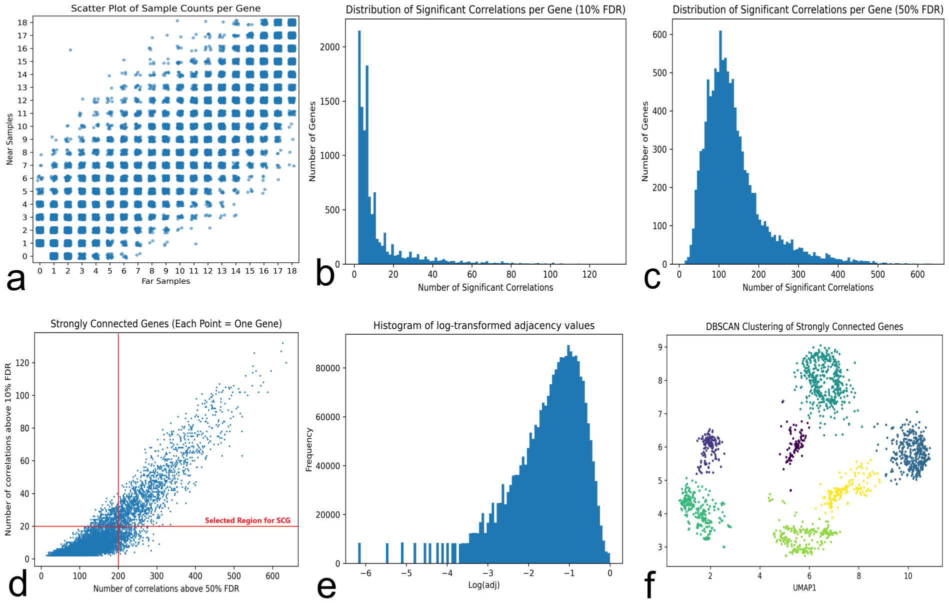

**Fig. 2 Visualization of gene coexpression analysis and clustering for NCBI PRJNA1089183 dataset** [17] (a) Relationships between gene expressions in two sets of samples: "near samples" and "far samples". The x-axis indicates the number of samples in which genes are expressed under "far" locations, whereas the y-axis reflects the number of samples in which the same genes are expressed under "near" locations. The genes expressed in only a few samples (both "near" and "far") are clustered near the bottom left, whereas genes expressed in many samples are clustered toward the top right. (b) Distribution of the number of correlations with FDRs less than 10%. (c) Distribution of the number of correlations with FDRs less than 50%. Both graphs are right-skewed, but in the 50% FDR histogram, the genes show a broader distribution of correlations. (d) Scatter plot showing the relationship between the number of correlations for above 10% FDR (correlation threshold: 0.81) and the 50% FDR (correlation threshold: 0.64) for each gene. The data points follow a linear pattern, which indicates that genes with a high number of significant correlations at 50% FDR ($\approx$ up to 600 correlations) also have a high number at 10% FDR (with a maximum of $\approx$ 120 correlations). The dense clustering in the lower left indicates that many genes have fewer than 200 connections for both criteria. The distribution was used to identify the selection of genes that maintain significant correlation even as the FDR threshold increased. Genes with $\geq$200 correlations at 50% FDR and $\geq$20 at 10% FDR were selected as strongly connected genes (SCGs) for downstream analysis. (e) Histogram of log-transformed adjacency values representing the strength of gene correlations. (f) UMAP visualization of density-based clustering of strongly connected genes, with 7 distinct clusters labelled by color. Genes that show similar differential coexpression patterns are clustered together.

pairs show moderate connectivity changes, a smaller subset of genes experiences significant shifts in coexpression, which is important for network analysis and identification of biomarkers of device-tissue interactions.

In Figure 3, we plotted the clustering plots for the top 9 clustering parameters based on the composite scores of the Silhouette and Davies-Bouldin index scores. Based

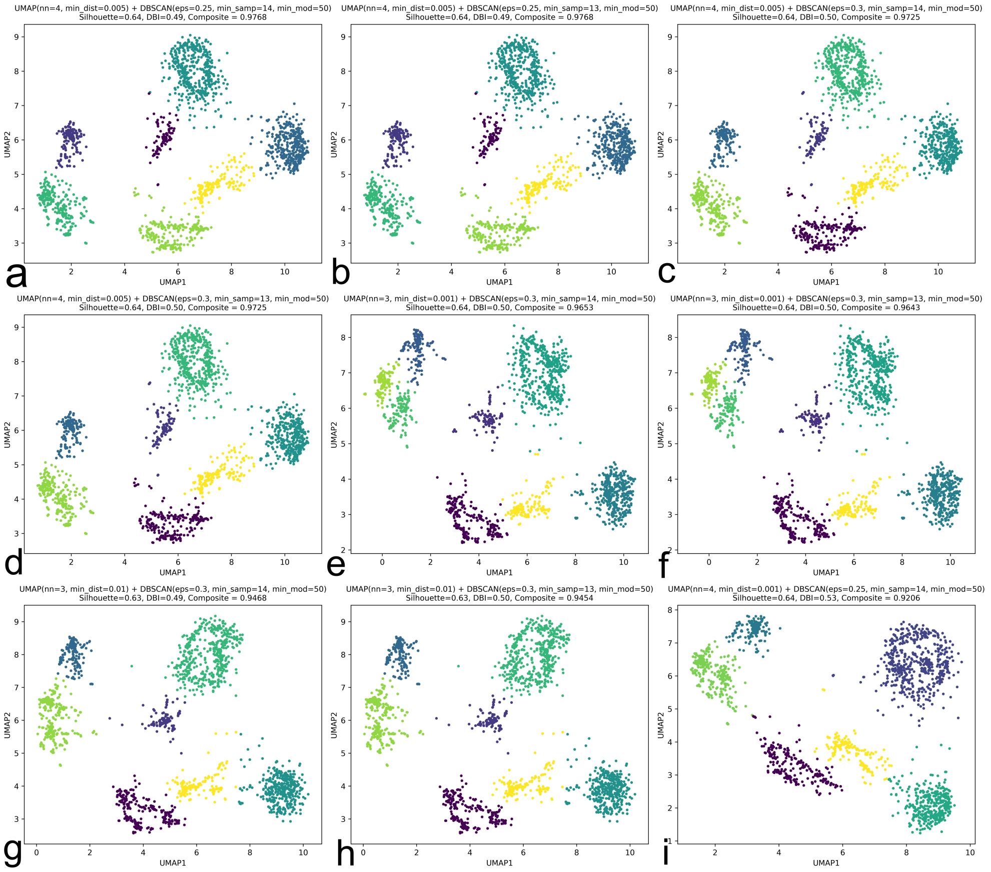

**Fig. 3 Visualization of clustering results for top nine parameter configurations of UMAP and DBSCAN** The plots are evaluated using a composite score of Silhouette Score and Davies-Bouldin Index (DBI). Each plot represents a unique combination of UMAP parameters (number of neighbors, minimum distance) and DBSCAN clustering parameters (eps, minimum samples). The silhouette score and the DBI scores are displayed in the plot titles. We selected figure (a) as the optimal clustering cluster.

on figure 3(a), there are 7 clusters. While several sets of parameters yielded a similar number of modules, we ultimately chose the one that balanced cluster quality (in measures like the silhouette score and Davies-Bouldin index), biological interpretability, the highest number of nearest neighbors, and the minimum sample. Also, it ranked top among all the clustering parameters in our grid search. We adopted this approach to reduce noise, provided it does not overmerge different subgroups. We found the hub genes for each module mentioned in Table 1.

We have generated the correlation heatmap for this dataset, which is described in Figure 4. It shows how gene co-expression patterns change with distance from the implanted electrode. By splitting correlations into near-device (upper triangle) and far-device (lower triangle) regions, it appears that specific gene modules behave differently across spatial contexts. Modules 1 and 7 show a strong positive correlation within themselves for both near and far regions. But there are not as significant changes in the correlation of the near-device samples to the far-device samples. Modules 2 and 5 show strong internal correlation only in the near-device region, suggesting these modules are locally activated. Modules 3 and 4 show a strong internal correlation only in the far-device region.

To assess our pipeline, we used two approaches: (1) we tested our pipeline on a dataset assessed with the original DiffCoEx approach, and (2) we applied an alternative coexpression analysis package to our data and compared the list of resulting hub genes. For our first approach, we tested our method on a dataset (Gene Expression Omnibus GEO GSE5923 - Eker dataset) that contains Affymetrix gene expression profiles of the renal cortex outer medulla in wild-type and Eker rats treated with carcinogens. We used the filtering method described in [15] alongside our computational methods to find the clusters and the hub gene list. We determined the number of correlations above 10% and 50% FDR (Figure 5) and then determined the number of strongly connected genes (Table 2). In this table, we have introduced three different correlation criteria - strict, moderate, and lenient - based on the number of correlations for building the network, which eventually influences its structure. Strict thresholds require high values (600) for the number of correlations, forming only the strongest and most direct gene relations. This threshold helps detect the core functional modules. The moderate number of correlations (400) balances the strongest and weakest correlations. The lenient number of correlations threshold includes a lower value of the number of correlations (200) between the strongly connected genes, which may

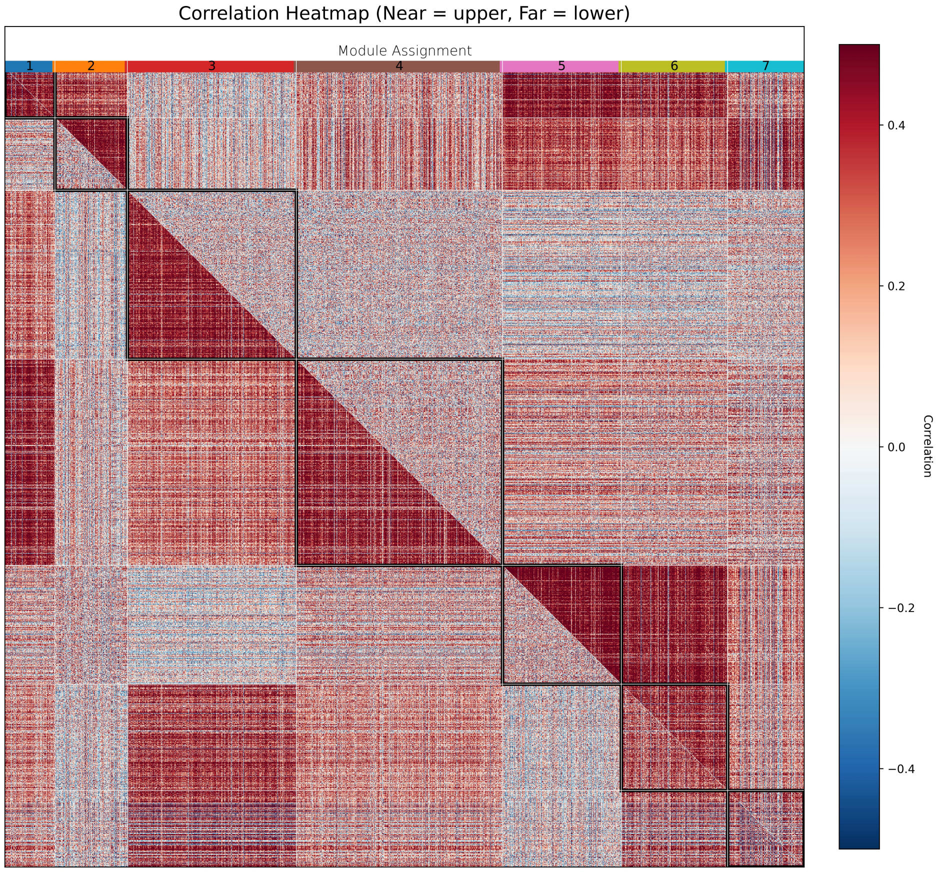

**Fig. 4 Correlation Heatmap of Gene Modules (Near vs. Far from the Implant Location)** The upper triangle of the heatmap shows the gene–gene correlation values in near-device samples, while the lower triangle shows correlations in far-device samples for the NCBI SRA PRJNA1089183 dataset. Each block along the diagonal represents a co-expression module, and off-diagonal blocks indicate interactions between different modules. Red colors represent stronger positive correlations, and cooler blues represent negative correlations. By comparing the upper and lower halves, we can observe how gene co-expression patterns shift depending on the distance from the implant, which is a key aspect of differential co-expression.

form compact and dense networks by including weak and noisy correlations. According to our experiment, at a higher 50% FDR (correlation threshold: 0.31), the number of correlations threshold for the strict criterion increased to 3500, though we got only 8.1% genes as significant. This indicates that when we allowed for a higher rate of false positives, a higher correlation value was needed to still be considered statistically

**Table 1** List of Hub Genes with Cluster Labels and Functions for Our Dataset. The list of genes with their ranks is given in the Supplementary Files S1

| Cluster Label | Name of the Hub Gene | Function | Hypothesized Relevance to the Brain Implant |
|---|---|---|---|
| 1 | **TRMT1L** | RNA methyltransferase; regulates tRNA dimethylation and stress response | Involved in oxidative and ER stress responses induced by surgical trauma and foreign body response during implantation. |
| 2 | **CACNB1** | Beta subunit of voltage-dependent L-type calcium channels; modulates calcium kinetics | Important for neuronal excitability and synaptic plasticity; activated by mechanical injury and calcium-dependent stress pathways surrounding the implant. |
| 3 | **RPS4X**, ATP6V0E1 | Ribosomal protein supporting translation recovery; ATP6V0E1 acidifies lysosomes for autophagy and membrane repair | Coordinates translation recovery and autophagic clearance essential for cellular survival after brain implant injury. |
| 4 | **RNF10** | E3 ubiquitin ligase; synapto-nuclear messenger regulating transcription | Coordinates transcriptional programs essential for dendritic spine remodeling and neuronal plasticity after synaptic injury from implantation. |
| 5 | **TRIM25**, SLC49A4 | E3 ubiquitin ligase activating RIG-I immune signaling; SLC49A4 supports antioxidant defense via pyridoxine transport | Activates sterile inflammation pathways and supports antioxidant defense after implantation-induced ROS generation. |
| 6 | **VASH2**, HPS4 | Promotes angiogenesis and endothelial cell migration; HPS4 regulates vesicle trafficking and debris clearance | Coordinates vascular remodeling and intracellular quality control critical for tissue recovery after brain implantation. |
| 7 | **TM7SF3**, SNRK | ER stress modulator reducing proteotoxic stress; SNRK regulates metabolism and suppresses inflammation | Integrates ER stress reduction, metabolic homeostasis, and anti-inflammatory defenses supporting survival after implantation injury. |

significant. Because of this, we considered the 50% FDR to be less efficient for our goal of identifying reliable connections. Therefore, we selected the strict criterion with a 10% FDR (correlation threshold: 0.513), utilizing the lower number of correlations threshold required for significance at this stricter FDR, to ensure that our analysis focused on the most confident gene-gene interactions, enhancing the reliability and

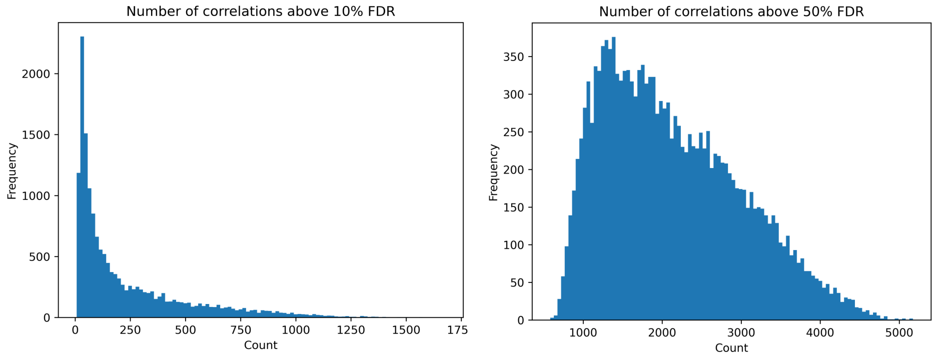

**Fig. 5 Distribution of Gene Correlations in GEO GSE5923 Dataset.** Number of correlations above 10% FDR (Left) and 50% FDR (Right) for the Gene Expression Omnibus GEO GSE5923 dataset [16]

biological relevance of the identified co-expression modules. This kind of threshold-based adjustment of SCGs is common for cancer datasets due to their complexity and heterogeneity [34].

**Table 2** Number of Strongly Connected Genes for 10% and 50% FDR for three different criteria for Eker Dataset [15]. The total number of genes after filtration was 15922.

| | **10% FDR** | | | **50% FDR** | | |
|---|---|---|---|---|---|---|
| **Selection** | **No. of Corr. for SCG** | **No. of SCG** | **Percent of SCG** | **No. of Corr. for SCG** | **No. of SCG** | **Percent of SCG** |
| Strict | 600 | 1813 | 11.4% | 3500 | 1295 | 8.1% |
| Moderate | 400 | 3248 | 20.4% | 2000 | 7888 | 49.5% |
| Lenient | 200 | 5884 | 37.0% | 1200 | 13612 | 85.5% |

Figure 6 shows the UMAP clusters for the Eker dataset [16]. It is evident that the clusters with a minimum sample size of 10 and a minimum distance of 0.01 prominently included five clusters (Figure 6 (b)). These clusters were chosen based on their overlap and distance from one another. We identified 5 different hub genes from these clusters, which are listed in Table 3.

For our second approach, we applied the GWENA package [33] to analyze gene coexpression networks and compare the results with our data. The input consisted of

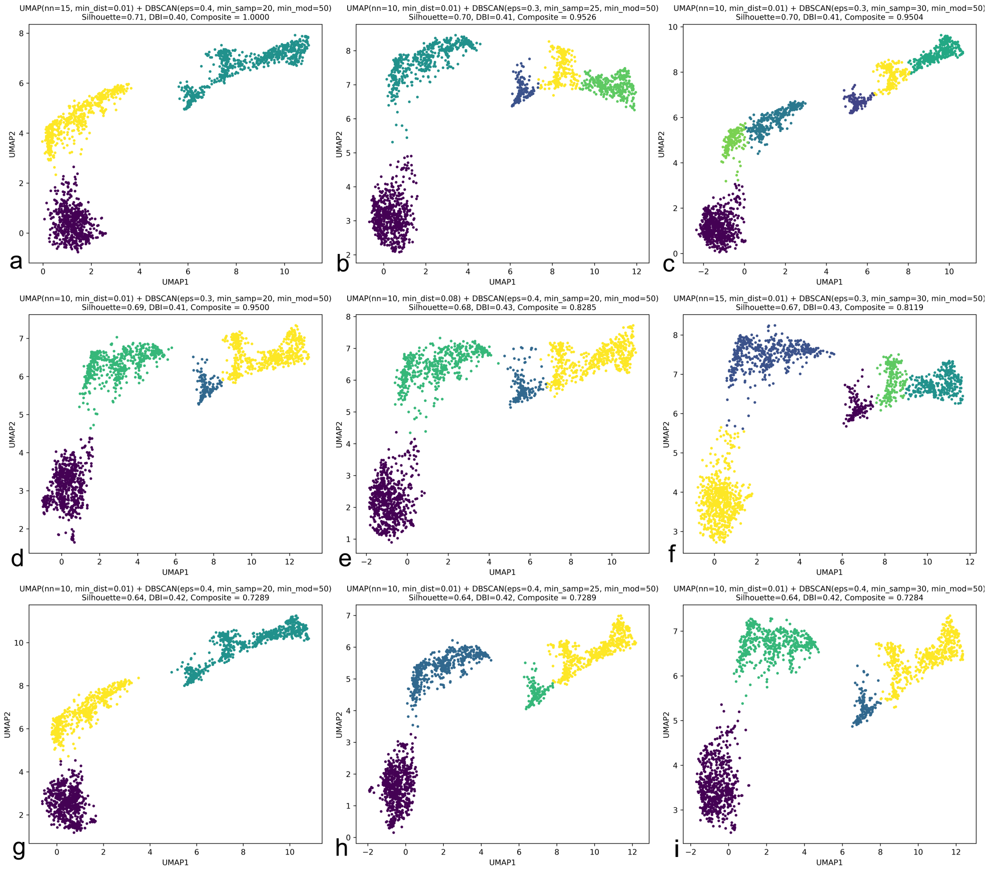

**Fig. 6 Density-based clustering of strongly connected genes visualized via UMAP embedding.** Each plot represents clustering results under different parameter settings for UMAP and DBSCAN for the Eker Dataset.

the preprocessed gene expression data from our dataset. Similar filtering steps were implemented to remove the genes with low expression counts and low variance, ensuring that only biologically relevant genes were accepted for network construction. As the package had no FDR thresholding technique, the number of genes remaining after applying the filtration techniques was greater than that in our analysis. GWENA uses Spearman correlation, and the network construction process utilizes a modified version of WGCNA, where a correlation matrix is first generated, followed by an adjacency matrix, and finally, a topological overlap matrix (TOM) to ensure biologically relevant coexpression relationships. Then, module detection was performed via hierarchical clustering, where the modules were adjusted through dynamic tree cutting and merged based on eigengene similarity. To obtain the hub genes, the authors computed key network centrality metrics, including the highest connectivity, highest degree, and Kleinberg score. GWENA produced seven distinct modules, which we compared to our results.

**Table 3** Hub genes for the Eker Dataset [16] and comparison with the original paper [15]

| **Module** | **Hub Genes** | **Function** | **Relevance to the Original Paper's Result** |
|---|---|---|---|
| 1 | **MRPS26** | Involved in mitochondrial protein synthesis and maintaining mitochondrial function. | Blue Module (Metabolic activity and mitochondrial function). |
| 2 | **PXMP2** | Facilitates the exchange of small solutes critical for ROS detoxification. | Green Module (Oxidation-reduction processes). |
| 3 | **ADCY6** | Plays a role in cAMP-mediated synaptic plasticity and neuronal survival; improper cAMP regulation is linked to cancers. | Yellow Module (Cancer-associated pathways). |
| 4 | **GPR12** | Related to cancer via GPCR-mediated control of proliferation, cell adhesion, and survival. | Yellow Module (Cancer-associated pathways). |
| 5 | **CD40LG** | Mediates immune signaling; involved in cancer biology, tumor progression, and metastasis formation. | Red Module (Cancer-related pathways). |

## 4 Discussion

Spatial transcriptomics has been developed as a revolutionary method for delineating gene expression within the spatial context of tissues, offering vital insights at the electrode-tissue interface in neural implants [35]. By visualizing RNA transcripts around implanted electrodes, we can understand cellular responses such as neuronal activation, inflammation, and gliosis. Identifying individual genes of interest from the

**Table 4** Hub Genes identified for our dataset by applying GWENA package [33]

| Module | Gene | Function | Hypothetical Relevance |
|---|---|---|---|
| 1 | **SPATS2** | Involved in cell cycle regulation. | May contribute to glial proliferation after implantation injury but has less primary relevance to acute injury. |
| 2 | **PINX1** | Involved in telomere maintenance. | Likely more relevant to chronic stress conditions rather than acute injury response. |
| 3 | **PDCD2l** | Associated with apoptosis regulation. | Corresponds to the cell death pathways initiated by implantation injury. |
| 4 | **NDC80** | Involved in active cell division. | May contribute to cell proliferation after injury but less relevant in immediate stress response. |
| 5 | **UBTD1** | Associated with ubiquitin-protein ligase activity for protein degradation. | Essential for clearing damaged proteins after injury, supporting proteostasis and lysosomal degradation pathways. |
| 6 | **SLC49A4** | Supports antioxidant defense and neurotransmitter synthesis via pyridoxine transport. | Vital for managing oxidative stress and metabolic recovery after implantation-induced ROS generation. |
| 7 | **HCFC2** | Transcriptional coactivator involved in innate immune response. | Contributes to sterile inflammation triggered by device implantation, consistent with activation of inflammatory pathways. |

large lists of differentially expressed genes provided by this high-throughput technique poses a practical challenge [36]. To discover meaningful patterns, modern computing methods and rigorous statistical analyses are needed because of the large amount of data, biological complexity, and potential for false positives. Integrating RNA sequencing data with other modalities and applying validation procedures are crucial for confirming the significance of the identified genes. Various methods of analysis have been used to solve these difficulties.

The successful integration of neural implants into the brain requires a thorough understanding of how diverse cell types respond at the molecular level. Neurons, astrocytes, microglia, and oligodendrocytes all play diverse roles in responding to external stimuli. Identifying the hub genes that regulate these responses can help optimize implant design and reduce adverse consequences. TRMT1L, the hub gene in module 1, is required for the dimethylation of tRNA-Tyr-GUA. A tRNA methyltransferase modifies tRNA-Tyr-GUA by adding a dimethyl group to the guanine base at position

27, resulting in the m2,2G modification. They are also sensitive to oxidative and endoplasmic reticulum (ER) stress [37]. The observation of disrupted motor coordination in the absence of TRMT1L indicates its involvement in neurological functions [38]. This is particularly relevant to our experiment related to the brain implants because the implantation process induces cellular stress in the surrounding neural tissue through surgical trauma, foreign body response, and homeostatic disruption.

The hub gene for module 2, CACNB1, encodes the beta-1 subunit of voltage-dependent L-type calcium channels. This subunit influences the calcium channel's voltage dependence and kinetics, hence regulating calcium inputs into cells [39]. These L-type calcium channels, particularly those with CACNB1, are important for neuronal excitability and synaptic plasticity [40]. Brain implantation will cause mechanical injury, and the cells are physically damaged, and the BBB (blood-brain barrier) is breached. So, there will be abnormal depolarization as the neurons near the implant site become stressed [41]. Mechanical damage, like the electrode implantation, will activate global calcium-dependent stress pathways as seen in other types of brain injuries like TBI (traumatic brain injury)[42].

The hub gene for module 3 is RPS4X, which is a ribosomal protein, specifically part of the small 40S ribosomal subunit, essential for the recovery of protein synthesis machinery after cellular stress and injury. It plays a role in translation initiation and ribosome assembly [43]. Restoration of translation capacity is important for neuronal and glial cells to resume the synthesis of proteins critical for repair. This gene has also emerged as a hub gene in traumatic brain injury [44]. We also found ATP6V0E1, playing a key functional role in this module by encoding a subunit of the vacuolar ATPase (V-ATPase) complex, which acidifies intracellular organelles [45], a requirement for efficient lysosomal degradation, autophagic flux, and membrane repair. ATP6V0E1 controls the pH environment where these pathways operate.

RNF10 emerged as the hub gene for module 4, which is an E3 ubiquitin ligase and synapto-nuclear messenger that relays signals from the synapse to the nucleus [46]. Electrode implantation in the brain causes mechanical disruption of synaptic connections, which triggers the activation of nuclear signaling pathways. RNF10 translocates to the nucleus in response to this disruption [47]. Upon translocating to the nucleus, RNF10 regulates the transcription of immediate-early genes like Arc [48] and Egr1 [49]. These genes are important for processes like dendritic spine remodeling, which is essential for neuronal repair and plasticity. Because of its role in coordinating transcriptional programs related to synaptic plasticity and stress adaptation, it suggests that its expression will be highly correlated with injury-responsive genes, thus making it a sensible candidate for a hub gene.

The hub gene for module 5 is TRIM25, which is known as a multifunctional E3 ubiquitin ligase and is involved in RNA metabolism and controls the activation of RIG-I, which triggers the innate immune response, even from sterile inflammation [50][51]. After brain implant insertion, sterile inflammation is triggered by damage-associated molecular patterns (DAMPs) released from injured tissue. DAMPs stimulate RIG-I-like receptors (RLRs) and trigger the upregulation of inflammatory signaling cascades (NF-$\kappa$B, IRF3) [52][53][54]. This makes many stress or inflammation genes (IL1$\beta$, TNF$\alpha$, NFKBIA, etc.) activated [54], and TRIM25 likely correlates with these. The SLC49A4 gene emerged as a top gene for this module, which is also known as Disrupted in Renal Carcinoma 2 (DIRC2) and functions as a lysosomal pyridoxine (vitamin B6) transporter [55]. It exports essential vitamins into the cytoplasm, facilitates neurotransmitter synthesis, and supports amino acid metabolism and antioxidant defense in the brain [56][57]. After the microelectrode implantation, cellular and oxidative stress can occur in the cells of the brain and can produce ROS (reactive oxygen species) from damaged mitochondria and others [58]. So, this gene is expressed because it works as the supplier of pyridoxine, which is needed for antioxidant defense and neurotransmitter release.

VASH2, the hub gene for module 6, encodes vasohibin-2, a secreted protein with pro-angiogenic activity. Specifically, VASH2 promotes endothelial cell migration and proliferation, both essential processes in the formation of new blood vessels [59]. So, this activity is important for restoring perfusion to hypoxic and mechanically injured brain regions around the implant [60]. HPS4, a top gene in module 6, is a component of the Biogenesis of Lysosome-related Organelles Complex-3 (BLOC-3) and plays an important role in regulating endosomal-lysosomal trafficking under stress conditions [61]. After the brain implant, the device causes plasma membrane disruption, and the debris of the cells accumulates near the implant site, which triggers the endosomal and lysosomal clearance pathway [62]. Any disruption of endosomal-lysosomal function leads to debris accumulation, increased neuroinflammation, and promotes neurodegeneration [63]. So, HPS4 gene expression is related to the regulation of multiple injury-induced pathways, including vesicle trafficking and inflammation. While VASH2 drives vascular repair, HPS4 ensures intracellular quality control and debris removal. Together, these genes coordinate critical aspects of tissue recovery after injury.

In the last module, module 7, we found TM7SF3 as the hub gene with SNRK among the top genes. TM7SF3 encodes a transmembrane protein that regulates the unfolded protein response (UPR) and endoplasmic reticulum (ER) stress, both of which are required for cellular survival after injury. By helping to maintain proper protein folding and reducing proteotoxic stress [64], TM7SF3 maintains membrane and organelle integrity, which is essential after mechanical disruption caused by device insertion [65]. In addition, SNRK is a serine/threonine kinase that belongs to the adenosine monophosphate (AMP)-activated protein kinase (AMPK) family, which regulates metabolic activities in several cell types. This is an important regulator of cellular metabolic homeostasis [66]. In addition to its metabolic role, SNRK suppresses NF-$\kappa$B signaling, decreasing the synthesis of inflammatory cytokines [67]. SNRK expression is likely to correlate with a wide range of metabolic, anti-inflammatory, and stress-response genes activated by brain injury after implantation. TM7SF3 protects cellular machinery under stress, while SNRK ensures energy preservation and reduces inflammation. This coordinated response is essential for reducing the impact of the initial injury and creating an environment favorable to tissue repair and functional recovery after brain implantation.

As the verification step, we applied our algorithm to the Eker dataset [16] to identify functionally relevant gene modules and their hub genes. Below, we describe the results of our analysis and compare them with the modules identified in the original study [15]. The initial difference study [15] revealed eight unique differentially expressed modules: black, blue, brown, green, orange, purple, red, and yellow. These modules encompassed approximately 1,800 genes. However, only four of these modules exhibited significant functional enrichment, consistent with the major biological responses expected after brain implantation. Specifically, the blue module was enriched for genes associated with mitochondrial function and lysosomal pathways. The green module was linked to oxidative stress response and mitochondrial metabolic processes. The yellow module showed enrichment for pathways related to protein localization, cell projection, and small GTPase-mediated signaling. The red module was primarily associated with cancer-related pathways, including endometrial, pancreatic, and renal cell carcinoma.

Our analysis identified five key modules, with hub genes corresponding to distinct biological functions. Module 1 (MRPS26) encodes a mitochondrial ribosomal protein S26, essential for mitochondrial protein synthesis and function [68], thus aligning with mitochondrial processes. Module 2 (PXMP2) encodes a peroxisomal membrane protein involved in transporting small solutes across the peroxisomal membrane, a crucial step in managing oxidative stress and ROS detoxification [69, 70], aligning it with oxidation-reduction processes. Modules 3 and 4 (ADCY6 and GPR12) are associated with cancer-related pathways [71, 72, 73, 74]. ADCY6 encodes adenylyl cyclase 6, which plays a role in cAMP-mediated synaptic plasticity and neuronal survival, and its signaling pathways are frequently dysregulated in cancer. GPR12 encodes a G protein-coupled receptor involved in proliferation, cell adhesion, and survival, all key processes in oncogenesis. Module 5 (CD40LG) encodes the CD40 ligand, a critical mediator of immune signaling with established involvement in cancer biology [75], particularly in stimulating tumor progression, growth, and metastasis. These modules exhibit significant overlap with functional themes from the DiffCoEx study [15], validating the differentially coexpressed gene networks.

In the next verification step, we applied the GWENA package [33] to our RNA-sequencing dataset and discovered the hub genes produced by it, as shown in Table 4. GWENA software and our method of analysis identified a similar number of coexpression clusters but yielded different hub genes, indicating key technical differences. The functions of the hub genes and their relevance to our results are provided in the Supplementary File S4. GWENA, which uses WGCNA for clustering, favors module preservation and connectivity within predetermined coexpression structures. In contrast, our approach uses DiffCoEx [15] to detect differential coexpression, allowing us to identify genes whose interactions change as a result of implantation rather than just those that remain significantly coexpressed. Furthermore, our hub gene selection method incorporates multimetric centrality analysis (degree, betweenness, eigenvector, and closeness centrality), whereas GWENA ranks genes only by degree and connectedness. As a result, GWENA's hub genes indicate generalized network stability. In contrast, our technique identifies functionally active genes in response to the brain implant, presumably making them more useful for investigating implant-induced molecular changes.

In this study, we implemented an optimized differential co-expression (DiffCoEx)-based clustering method to identify biologically relevant gene modules that show condition-specific co-expression changes. By integrating UMAP-based dimensionality reduction with DBSCAN clustering, we enhanced the ability to detect gene modules with distinct co-expression patterns. Our approach successfully identified hub genes that may serve as key regulators of biological responses, particularly in the context of neural implants.

## 5 Declarations

### 5.1 Ethics approval and consent to participate

We confirm that all methods and surgical procedures conducted in this study were authorized by the Michigan State University Animal Care and Use Committee.

### 5.2 Consent for publication

Not applicable.

### 5.3 Availability of data and materials

The dataset used in this study is available at NCBI SRA PRJNA1089183 and is provided under the NCBI Data Usage Policy. The code for this project can be found in our GitHub Repository (https://github.com/msureil/DiffCoRank) under the MIT license. The supplementary files include:

- Supplementary File S1: List of modules 1 to 7 genes for the NCBI SRA PRJNA1089183 Dataset, including the rankings.
- Supplementary File S2: List of top 6 genes in each module for the NCBI SRA PRJNA1089183 Dataset, including the hub genes in each module.

- Supplementary File S3: List of the function of the top 6 genes in each module for the NCBI SRA PRJNA1089183 Dataset, including the hub genes in each module.
- Supplementary File S4: List of hub genes and their functions for the NCBI SRA PRJNA1089183 dataset by using the GWENA software package.

#### 5.4 Competing interests

The authors declare no competing interests.

#### 5.5 Funding

This work was funded by an NIH RO3 award (NS127192).

#### 5.6 Authors' contributions

MM and EP conceived the study. AC and MM designed the study. AC, EP, and MM wrote the main manuscript text. AC prepared figures 4-6, while MM prepared figures 1-3. AC and MM conducted all the analyses. EP, MM, and AC interpreted the results. All authors reviewed the manuscript.

#### 5.7 Acknowledgements

The authors thank Dr. Cort Thompson for his helpful discussions and role in collecting the original data set. Dr. Mark Reimers also provided feedback on the approach.

## Supplementary Files

This is a list of supplementary files associated with this preprint. Click to download.

- SubmissionSupplementaryFiles.zip
